# Correction: Blood Pressure Control with a Single-Pill Combination of Indapamide Sustained-Release and Amlodipine in Patients with Hypertension: The EFFICIENT Study

**DOI:** 10.1371/journal.pone.0098699

**Published:** 2014-05-23

**Authors:** The PLOS ONE Staff

## Notice of Republication

This article was republished on April 30, 2014, to include figures in the main article that were previously published as Supporting Information. Please download this article again to view the correct version. The originally published, uncorrected article and the republished, corrected article are provided here for reference.

## Supporting Information

File S1 **Originally published, uncorrected article.**
(PDF)

File S2 **Republished, corrected article.**
(PDF)
