# Supplementary material for: Blood Pressure Control with a Single-Pill Combination of Indapamide Sustained-Release and Amlodipine in Patients with Hypertension: The EFFICIENT Study
Source: PLoS One. 2014 Apr 8;9(4):e92955. doi: 10.1371/journal.pone.0092955 (PMC3979648; doi:10.1371/journal.pone.0092955)
Supplement: Protocol S1 — Trial Protocol. (PDF) [file pone.0092955.s001.pdf]

CLINICAL TRIAL PROTOCOL

Study title:

Efficacy and Tolerability of Fixed Dose Combination of Indapamide S.R. (1.5 mg) + Amlodipine (5 mg) in patients suffering from stage II/III hypertension

Protocol/ Study number, and protocol version number and date:

SER/01/2009; Version 4.0 dated 10th Nov'09

IND name/number of the investigational drug:

Not applicable

Sponsor:

Serdia Pharmaceuticals (India) Pvt. Ltd.,
Serdia House, Off Dr. S.S. Rao Road,
Parel, Mumbai 400 012
Tel no.: 022-2416 2727; Fax no.: 022-2416 3171

Trial monitoring:

Clinical Research Department, Serdia Pharmaceuticals (India) Pvt. Ltd., Mumbai

Contact persons:

Dr. Ajaykumar Yadav, Clinical Project Manager
Dr. Nishant Sangole, Clinical Project Leader

Appendix-I: Declaration of Helsinki

Appendix-II: List of investigators

Center number: I__I__I

INDEX

S. No	Title	Page No.
1.	Introduction	3
2.	Study rationale	3
3.	Study objectives	3
4.	Study design	4
5.	Study population	7
6.	Eligibility criteria	7
7.	Study conduct	8
8.	Study treatment	8
9.	Forbidden medication	8
10.	Other concomitant medication	9
11.	Adverse events	9
12.	Ethical requirements	10
13.	IRB/IEC	11
14.	Study monitoring and supervision	11
15.	Drug supplies and management	11
16.	Biometry and statistical analysis	12
17.	Data archival	12
18.	Confidentiality	13
19.	Early study termination	13
20.	Study time table	13
21.	References	13
22.	Study at a glance	14
23.	Patient activity chart	14
24.	Signed agreements of protocol	15

1. Introduction

Optimum management of control of hypertension requires a multi-disciplinary approach including appropriate medication and compliance. Thiazide type diuretics, β blockers, Angiotensin Converting Enzyme inhibitors (ACEIs), Calcium channel blockers (CCBs) are established classes of antihypertensive drugs that are used as monotherapy or as fixed dose combinations (FDC).

However, there exists a population of patients who would require more than a single drug to achieve the target blood pressure control. A two-drug combination for most of the patients with stage 2 hypertension (moderate to severe hypertension) has been recommended by JNC 7 guidelines which include thiazide-type diuretic and ACEI or ARB or BB or CCB.[1, 2]

2. Study Rationale

Fixed dose combination (FDC) of Indapamide SR + Amlodipine is a new FDC for the Indian market and this proposed study is a part of the marketing approval review process in India. Indapamide SR and Amlodipine are available in the Indian market for past approximately 20 years and are individually marketed for the treatment of hypertension. They are also taken simultaneously to achieve target blood pressure control. For the first time, a pharmaceutically stable formulation is developed by the applicant for the purposes of compliance in the treatment; the efficacy and safety of this formulation in the clinical setting is planned to be evaluated through this phase III registration clinical trial.

3. Study Objectives

3.1 Primary Objective

The primary objective of the study is to assess the blood pressure reduction rates in moderate to severe hypertensive patients after treatment with an FDC (Indapamide S.R. 1.5 mg + Amlodipine 5 mg) given once daily in the morning during the study evaluation period.

3.2 Secondary Objectives

To access the safety and tolerability of Indapamide S.R. 1.5 mg + Amlodipine 5 mg

4. Study Design

4.1 A multicentric, open, non-comparative study

4.2 Study flow-chart

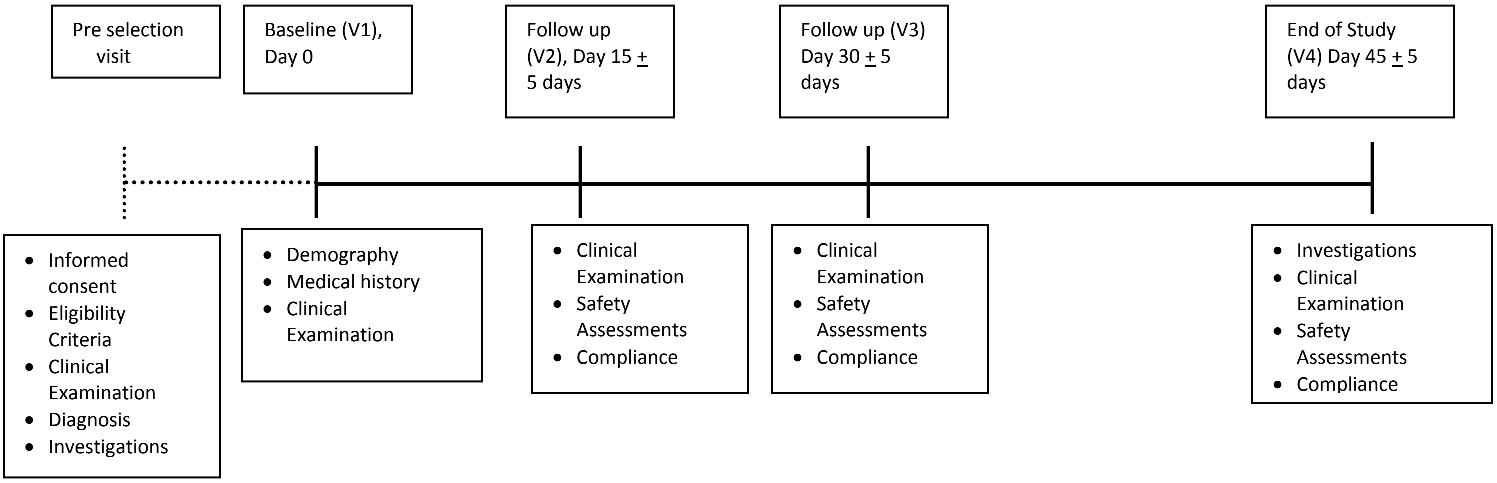

4.3 Study Procedures

The trial will be carried at various select centres in India. It will be conducted in the Out-patient clinics by physicians.

The patients will need to undergo screening prior to their inclusion as stipulated in the protocol.

4.4. Study visits and assessments

4.4.1 Pre selection visit

1) After the clinical diagnosis of hypertension in the subject the investigator will take the medical history of the patient and shall perform the physical examination. If the subject

is found eligible, an informed consent for participation will be obtained and a baseline ECG and laboratory investigations will be performed.

2) The investigator will ensure that the subject fulfills the eligibility criteria as per the inclusion and exclusion criteria stipulated in the protocol to participate in the study.

4.4.2 Baseline Visit (Visit 1, Day 0)

At this visit following activities shall be performed:

Patient Demography, Medical History, Inclusion and Exclusion criteria, Clinical & vital examination and associated diseases, Current treatment for hypertension or any other concomitant medication, if applicable shall be documented on the case report form.

The blood pressure measurement at each visit shall be carried out as per the JNC VII guidelines as under:

For blood pressure measurement, the auscultatory method with a properly calibrated and validated instrument should be used. Patient should be seated quietly for at least 5 minutes in a chair (rather than on an examination table), with feet on the floor and arm supported at heart level. An appropriate-sized cuff (cuff bladder encircling at least 80 percent of the measuring arm) should be used to ensure accuracy. At least two measurements should be made.

The investigator shall provide the study medication to the patient for the next 15 days (± 5 days) and will give necessary instructions regarding the administration. The investigator shall emphasize to every patient to take the medication every morning at the same time. He shall also instruct the patient not to take extra doses in case the dose has been missed. The investigator shall also inform the patient to contact him in case of any doubt or discomfort experienced. The patients will be asked to bring back the remaining study medication during the next visit.

During the study, at every follow-up (f/u) visit, heart rate and blood pressure will be measured and Adverse Events (AEs) if any shall be recorded. After enrollment, the trial drug shall be dispensed to each of the participant (daily dose of one tablet per day in

morning with breakfast) till his next study visit and subsequently thereafter till end of study.

4.4.3 Follow up visit (Visit 2, Day 15 ± 5 days)

At the follow-up visit 2, the investigator shall conduct the clinical and vital examination of the patient. He shall check the compliance to the study medication and shall interview the patient for any undesirable effects experienced since the last visit. At each visit, the doctor will also confirm that each patient is complying with the prescription and not missing any doses. The unused study medicine shall be collected from the patient. The investigator shall dispense the medications for the next 15 ± 5 days to the patients.

4.4.4. Follow-up visit (Visit 3, Day 30 ± 5 days)

At the follow-up visit 3, the investigator shall conduct the clinical and vital examination of the patient. He shall check the compliance to the study medication and shall interview the patient for any undesirable effects experienced since the last visit. At each visit, the doctor will also confirm that each patient is complying with the prescription and not missing any doses. The unused study medicine shall be collected from the patient. The investigator shall dispense the medications for the next 15 ± 5 days to the patients. Clinical and vital examination, Compliance to study drug, AEs if any shall be recorded.

4.4.5 End of Study (Visit 4, Day 45 ± 5 days)

This is the end of study visit. The investigator shall conduct the clinical and vital examination of the patient. He shall check the compliance to the study medication and shall interview the patient for any undesirable effects experienced since the last visit. At each visit, the doctor will also confirm that each patient is complying with the prescription and not missing any doses. The unused study medicine shall be collected from the patient. Additionally laboratory investigations (section 4.4.6) and ECG shall be repeated at the end of study. The investigator shall prescribe the most appropriate medicine for the treatment of hypertension to the patient.

4.4.6 Laboratory Assessments

The laboratory investigations as under will be performed during screening and at end of study (Visit 4, Day 45± 5 days). All tests will be performed at laboratories associated with trial centers or otherwise defined before the initiation of the trial. 5 mL of blood and 30 mL of urine will be collected, each at visit 1 and visit 4 (total blood loss for each patient = not more than 10 mL)

The laboratory test will include -

Hematology	-	CBC, ESR
Biochemistry	-	Total Bilirubin, AST, ALT, Serum Creatinine, Blood urea, Fasting blood sugar, Serum Electrolytes, Lipid profile
Urine Analysis	-	Routine & microscopy
ECG	-	Chest

5. Study population

240 patients (30 patients per centre) will be included in the study satisfying the eligibility criteria as per the protocol, with an objective to obtain at least 200 patients completing the study.

6. Eligibility Criteria

6.1 Inclusion Criteria

1. Newly diagnosed untreated hypertensive patients having a baseline blood pressure $\geq$ 160/100 mmHg) OR
2. Uncontrolled patients of hypertension with a baseline blood pressure $\geq$ 140/90 mmHg on monotherapy with a Calcium Channel Blocker (CCB)
3. Patients of either sex

4. With confirmed diagnosis of Stage II/ III (moderate to severe) hypertension, as per JNC 7 guidelines

6.2 Exclusion criteria

Patients presenting with any of the following will not be included in study:

1) Uncontrolled diabetes
2) H/o myocardial infarction within the previous 3 months
3) H/o cerebrovascular event within previous 3 months
4) Uncontrolled arrhythmias
5) H/o Heart failure
6) Patients with severe impaired renal function serum creatinine levels > 5.3 mg/dl and / or serious liver disorders
7) Patients unwilling to give informed consent.
8) Patients with history of hypersensitivity to Indapamide or Amlodipine
9) Any contraindication to Thiazide type diuretic and/or CCB
10) Pregnancy or lactation
11) Patients with any other condition or disease, which in opinion of the investigator could preclude evaluation of response to study medication or hamper the safety of the trial participant.
12) Patients previously enrolled in similar trial in the last 6 months.

7. Study conduct

The study will be conducted as per the GCP guidelines and the “Ethical Guidelines for Biomedical Research on Human Participants” issued by ICMR and the other regulations in force in India.

8. Study treatment

Each eligible patient shall receive the study medication under evaluation from the participating investigator. The dose of this FDC is once daily in the morning.

9. Forbidden medication

Any other concurrent antihypertensive medication is forbidden during the study period.

10. Other concomitant medication

All other concomitant medicines as per the discretion of the investigator will continue except those that are contraindicated as per product fact sheet.

11. Adverse Events

All adverse events whether *expected* (as stated in the proposed product fact sheet) or *unexpected* (those not mentioned in the product fact sheet) reported by patients or observed must be recorded in the CRFs with information about severity i.e. whether

- Mild: no significant interference with usual activities;
- Moderate: significant interference with usual activities or;
- Severe: prevents usual activities, date of onset duration and action taken regarding study drug.

Relation of adverse events to study medication will be described as unrelated, probable or possible.

- Unrelated: a reaction that does not follow a reasonable temporal sequence from the administration of the medicine; is not known adverse reaction pattern of the suspected medicine; could have been produced by the patients clinical state, inter current illness, trauma, accidents etc.
- Probable: A reaction that follows the reasonable temporal sequence from administration of medicine; that follow as a known or expected response pattern to the suspected medicine; and that could not be reasonably explained by the known characteristics of the patient's clinical state
- Possible: reaction that follows the reasonable temporal sequence from administration of medicine; that follows as a know or expected response pattern to the suspected medicine; but that could readily have been produced by a number of other factors including the patients clinical state or other modes of therapy administered to the patients.

.

Any serious adverse effects must be notified immediately by telephone to the study monitor and subsequently in writing as specified later in this section.

A serious event or reaction (SAE) is any unto odd medical occurrence that at any dose:

- Results in death
- Is life threatening
- Requires inpatient hospitalization or prolongation of existing hospitalization
- Results in persistent or significant disability/incapacity, or
- Is a congenital anomaly/birth defect

Reporting of AEs

All serious adverse events whether considered to be associated with the study medication or not, must be reported within 24hrs, or at least on the following working day by telephone or fax (fax no. 022-2416 3171) to the sponsor. Subsequently the investigator should complete the serious adverse event form attach with the case report form and fax the same to the sponsor. The event must also be documented on the adverse event page on the case report form.

The investigator would also inform the ethics committee as soon as possible within 7 working days of it occurrence.

12. Ethical Requirements

Informed Consent

Informed consent will be obtained from each subject. The investigator will give each subject full information about the nature, meaning and importance of the study & a description of the procedures to be followed by the subject, all in accordance with Declaration of Helsinki. They will further be given a description of any foreseeable risks and discomforts. Subjects will also be told that they have the right to opt out of the trial at anytime without having to give reasons and without prejudice to further treatment. Each subject will be given a copy of the subject information sheet and will be given sufficient time to consider the implications of the study before deciding whether or not to participate in the trial.

The Informed consent form must be signed by the subject and the investigator. At this time the subject must have the legal capacity and be able to comprehend the nature, meaning, importance and risks of the study and to make up his/her mind accordingly.

If the subject is illiterate, subject's legally acceptable representative (LAR) may understand and sign the Informed consent form.

If the subject and subjects LAR both are illiterate an impartial witness (a person, who is independent of the trial and who cannot be unfairly influenced by people involved in the trial) will attend the entire informed consent process and will read out the subject information sheet and the informed consent form in a language best known to the subject.

Signed Informed consent means the acceptance that the individual's data will be known to the Investigator, the sponsor and possibly the Regulatory Authorities.

13. IRB/Ethics Committee

The study will be performed in accordance with the principles stated in the Declaration of Helsinki. Prior to study initiation, approval of the study protocol will be obtained from the IRB/EC. The opinion of the EC will be dated and given in writing. Whenever possible, the names and titles of the members attending the EC meeting should be appended. The approval must clearly identify the protocol and other documents submitted for reviews, by title and study code.

14. Study monitoring and supervision

The study will be managed and supervised by the Clinical Research Department of the sponsor. The responsible people will maintain the contact with the investigator and will visit the centres at the time of study initiation, during the study period and at the end of the study. They will verify all the documents and source data pertaining to this study at the study sites.

15. Drug Supplies and management

The study medication, FDC of Indapamide S.R.1.5 mg + Amlodipine 5 mg is manufactured by Serdia Pharmaceuticals (India) Pvt. Ltd. will be used in the study and supplied at the site. It is manufactured as per the current cGMP norms and the product specifications and the certificate of analysis shall accompany the product. It will be supplied in the sealed aluminium strips with appropriate labels and patient numbers. The study product shall bear the label "FOR CLINICAL TRIAL USE ONLY".

At each centre the study medication inventory shall be maintained and monitored. Every dispensing and return of unused study medication shall be documented. The unused study medication will be collected back by the sponsor and will be destroyed. Appropriate documentation shall be made for the same and shall be archived.

16. Biometry and statistical analysis

The data of the study will be statistically analysed using SPSS version 11 statistical programme using appropriate statistical tests.

16.1 Primary evaluation criteria: The primary evaluation criterion is the efficacy of the treatment in achieving the target blood pressure control. This will be evaluated as under:

- Reduction in blood pressure from baseline to end of study
- Blood pressure control rates observed in the patient population

16.2 Secondary evaluation criteria:
Safety and tolerance of this drug treatment in terms of side effects profile.

17. Data Archival

Copies of the CRFs, laboratory investigation reports, and other original source data related to the study will be preserved for 15 years by the Investigator. A signed inventory of the stored data will be held by the Investigator and a copy by the sponsor. The protocol, IB, CRFs copies of regulatory permission, drug analytical data and all correspondence and reports related to the study will be retained by Serdia Pharmaceuticals (India) Pvt. Ltd. for 15 years.

18. Confidentiality

The identity of patients and data generated in the study will be held in strict confidence. Data will be available only to doctors/scientists/auditors involved in the study and to the Regulatory Authorities.

19. Early Study Termination

The study will be terminated in case of any safety issues and /or protocol violations. The study may also be discontinued at a center for reason of particularly low recruitment rate, or inadequate data recording or any other issues that may necessarily require study termination. In any case, the investigator shall keep the Ethics Committee updated on the study progress. The sponsor shall keep the regulatory authorities informed about such decisions.

20. Study Time Table

Scheduled Date of Start : January 2010

Scheduled Date of completion : April 2010

21. References

1. The JNC 7 report. The seventh report of the Joint National Committee on Prevention, Detection, evaluation, and Treatment of high blood pressure. JAMA 2003; 289: 2560-72
2. NICE clinical guideline 34.Hypertension: management of hypertension in adults in primary care. Issue dated June 2006. www.nice.org.uk

22. Study at a glance

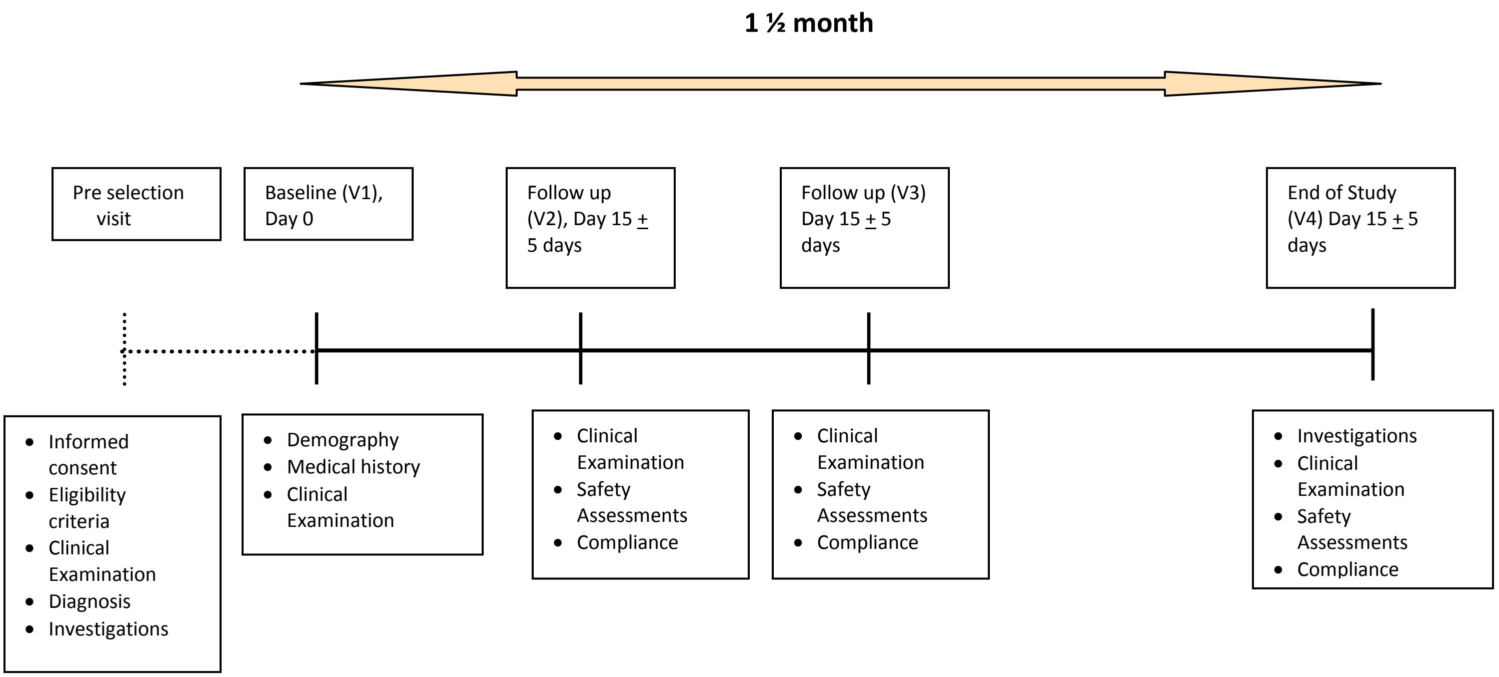

23. Patient Activity Chart

Activity	Pre Selection Visit	Baseline visit, Visit 1 (D_0)	Follow-up visit, Visit 2 (D_{15})	Follow-up visit, Visit 3 (D_{30})	End of study visit, Visit 4 (D_{45})
Informed Consent	X	--	--	--	--
Blood pressure, Heart rate	X	X	X	X	X
Laboratory investigations	X	--	--	--	X
Eligibility Criteria	X	X (based on lab reports)	--	--	--
Demography, clinical characteristics & Medical history	X	--	--	--	--
Clinical examination	--	X	X	X	X
Study medication to be provided	--	X	X	X	--
Dosage schedule of study medication	--	Once daily with breakfast	Once daily with breakfast	Once daily with breakfast	--
Adverse events	--	--	X	X	X
Treatment compliance	--	--	X	X	X

24. Signed Agreement of Protocol

Signature of the Investigator:

I have read the Clinical Study Protocol/ CRF specified below. I agree to undertake the study according to the procedures specified in the protocol. I also agree to provide Serdia Pharmaceuticals (India) Pvt. Ltd. with a report of the study including complete test results and all data developed at my centre. Being a multicentric study, the results from all centers will be collected and a comprehensive report prepared for registration of the product.

Sponsor: Serdia Pharmaceuticals (India) Pvt. Ltd.

Study code: SER/ 01/ 2009

Title of the Study: Efficacy and Tolerability of Fixed Dose Combination of Indapamide S.R. (1.5 mg) + Amlodipine (5 mg) in patients suffering from stage II/ III hypertension

Name of the Investigator	Signature	Date
Dr. Deepak Namjoshi		
