# Supplementary material for: Correction: Blood Pressure Control with a Single-Pill Combination of Indapamide Sustained-Release and Amlodipine in Patients with Hypertension: The EFFICIENT Study
Source: PLoS One. 2014 May 23;9(5):e98699. doi: 10.1371/journal.pone.0098699 (PMC4032339; doi:10.1371/journal.pone.0098699)
Supplement: File S1 — Originally published, uncorrected article. (PDF) [file pone.0098699.s001.pdf]

Blood Pressure Control with a Single-Pill Combination of Indapamide Sustained-Release and Amlodipine in Patients with Hypertension: The EFFICIENT Study

Uday Jadhav[1]*, Jagdish Hiremath[2], Deepak J. Namjoshi[3], Vinod K. Gujral[4], Kamlakar K. Tripathi[5], Mohammad Siraj[6], Paramesh Shamanna[7], Michel Safar[8]

1 MGM New Bombay Hospital, Navi Mumbai, Maharashtra, India, **2** Poona Hospital & Research Centre, Pune, Maharashtra, India, **3** Criti Care Multi Specialty Hospital & Research Centre, Mumbai, Maharashtra, India, **4** Specialty Clinics A, New Delhi, India, **5** Institute of Medicine Science, Banaras Hindu University, Varanasi, Uttar Pradesh, India, **6** Owaisi Hospital & Research Centre, Deccan College of Medical Sciences, Hyderabad, Andhra Pradesh, India, **7** Bangalore Diabetes Centre, Medisys Clinisearch India Pvt. Ltd, Bangalore, Karnataka, India, **8** Université Paris Descartes, Assistance Publique, Hôpitaux de Paris, Hôtel-Dieu Centre de Diagnostic et de Thérapeutique, Paris, France

Abstract

Objective: Despite antihypertensive treatment, most hypertensive patients still have high blood pressure (BP), notably high systolic blood pressure (SBP). The EFFICIENT study examines the efficacy and acceptability of a single-pill combination of sustained-release (SR) indapamide, a thiazide-like diuretic, and amlodipine, a calcium channel blocker (CCB), in the management of hypertension.

Methods: Patients who were previously uncontrolled on CCB monotherapy (BP≥140/90 mm Hg) or were previously untreated with grade 2 or 3 essential hypertension (BP≥160/100 mm Hg) received a single-pill combination tablet containing indapamide SR 1.5 mg and amlodipine 5 mg daily for 45 days, in this multicenter prospective phase 4 study. The primary outcome was mean change in BP from baseline; percentage of patients achieving BP control (BP<140/90 mm Hg) was a secondary endpoint. SBP reduction (ΔSBP) versus diastolic BP reduction (ΔDBP) was evaluated (ΔSBP/ΔDBP) from baseline to day 45. Safety and tolerability were also assessed.

Results: Mean baseline BP of 196 patients (mean age 52.3 years) was 160.2/97.9 mm Hg. After 45 days, mean SBP decreased by 28.5 mm Hg (95% CI, 26.4 to 30.6), while diastolic BP decreased by 15.6 mm Hg (95% CI, 14.5 to 16.7). BP control (<140/90 mm Hg) was achieved in 85% patients. ΔSBP/ΔDBP was 1.82 in the overall population. Few patients (n = 3 [2%]) reported side effects, and most (n = 194 [99%]) adhered to treatment.

Conclusion: In patients who were previously uncontrolled on CCB monotherapy or untreated with grade 2 or 3 hypertension, single-pill combination indapamide SR/amlodipine reduced BP effectively—especially SBP— over 45 days, and was safe and well tolerated.

Trial Registration: Clinical Trial Registry – India CTRI/2010/091/000114

Citation: Jadhav U, Hiremath J, Namjoshi DJ, Gujral VK, Tripathi KK, et al. (2014) Blood Pressure Control with a Single-Pill Combination of Indapamide Sustained-Release and Amlodipine in Patients with Hypertension: The EFFICIENT Study. PLoS ONE 9(4): e92955. doi:10.1371/journal.pone.0092955

Editor: Giuseppe Danilo Norata, University of Milan, Italy

Received August 12, 2013; **Accepted** February 26, 2014; **Published** April 8, 2014

Copyright: © 2014 Jadhav et al. This is an open-access article distributed under the terms of the Creative Commons Attribution License, which permits unrestricted use, distribution, and reproduction in any medium, provided the original author and source are credited.

Funding: A research grant was provided by Serdia Pharmaceuticals (India) Pvt. Ltd. The funders had no role in study design, data collection and analysis, decision to publish, or preparation of the manuscript.

Competing Interests: The authors have read the journal's policy and have the following conflicts of interest: the authors received honoraria, research grants, or both, from Servier or Serdia Pharmaceuticals (India) Pvt. Ltd. This study was funded by Serdia Pharmaceuticals (India) Pvt. Ltd. Co-author Paramesh Shamanna is employed by Bangalore Diabetes Centre, Medisys Clinisearch India Pvt. Ltd. There are no patents, products in development or marketed products to declare. This does not alter the authors' adherence to all the PLOS ONE policies on sharing data and materials.

* E-mail: drjadhav0512@gmail.com

Introduction

Elevated blood pressure (BP) is one of the most important risk factors for cardiovascular mortality [1], and BP lowering is associated with reductions in cardiovascular and renal outcomes [2,3]. BP lowering that leads to BP control, however, is achieved in less than a third of hypertensive patients [4]. Systolic blood pressure (SBP), in particular, is difficult to control in clinical practice [5]. SBP, which is a better predictor of cardiovascular risk than DBP, increases linearly from 30 years, while diastolic blood pressure (DBP) decreases from 50 years [6].

Initiating treatment with a single-pill combination of two antihypertensive agents has been shown to be significantly more effective and faster at controlling BP than using the same two agents in a sequential drug titration strategy [7,8]. International guidelines on hypertension recommend initiation of treatment with a single-pill combination in hypertensive patients with multiple cardiovascular risk factors, evidence of organ damage,

or grade 2 or 3 hypertension [9–11]. Antihypertensive treatment compliance is also significantly better with a single-pill combination than with a combination's components given separately [12]. In consequence, initiation of antihypertensive treatment with single-pill combinations is becoming more common.

The 2013 European guidelines on hypertension management give calcium channel blocker (CCB)/diuretic single-pill combinations preferred status based on promising results from randomized controlled trials, including VALUE (Valsartan Antihypertensive Long-term Use Evaluation) and FEVER (Felodipine EVEnt Reduction) [13–15]. This combination is a good option in hypertensive patients with low renin levels who are inadequately controlled by a renin-angiotensin-aldosterone system (RAAS) inhibitor [16]. The prevalence of isolated systolic hypertension is likely to increase as the proportion of elderly patients in populations around the world increases, so the need for therapeutic answers to elevated SBP is growing. Diuretics and CCBs have been found to be the most effective antihypertensive classes for SBP reduction, and the best agents in these classes in one meta-analysis of 10 818 patients were indapamide SR and amlodipine [17].

The first single-pill representative of this CCB/diuretic combination recently became available in Europe. Its individual components—indapamide sustained-release 1.5 mg (SR), a thiazide-like diuretic, and amlodipine 5 or 10 mg, a CCB—have been shown to reduce hypertension [17,18] and cardiovascular risk [2,19] in randomized controlled trials. In a recent meta-analysis of 160 000 hypertensive subjects, amlodipine and indapamide were two of the three antihypertensive agents to significantly reduce mortality [20], indicating the potential of this particular combination. To determine its clinical relevance, we describe a multicenter, prospective, phase 4 study, EFFICIENT (EFfects of a FIxed Combination of Indapamide sustained-release with amlodipine on blood prEssure iN hyperTension), which examines the effects of single-pill combination indapamide SR/amlodipine 1.5/5 mg on BP reduction, BP control, and adverse events in a primary healthcare setting [21].

Methods

The protocol for this trial and supporting TREND checklist are available as supporting information; see **Protocol S1** and **Checklist S1**.

Ethics statement

The study protocol was approved by the ethics committees of each participating center. Ethics committee approval was therefore obtained from the following organizations: Ethics Committee, MGM New Bombay Hospital, Mumbai (date of approval, 28 January 2010), Clinical Ethics Forum, Mumbai (16 February 2010), Ethics Committee, Faculty of Medical Sciences, Banaras Hindu University, Varanasi (3 March 2010), Clinical Ethics Forum, Mumbai (16 February 2010), Bangalore Central Ethics Committee, Bangalore (27 January 2010), Institutional Ethics Committee, Deccan College of Medical Sciences & Allied Hospitals, Hyderabad (12 January 2010), and Ethics Committee, Poona Hospital & Research Centre, Pune (3 April 2010). The study, which is publicly registered (CTRI No.: 2010/091/000114), complies with the Guidelines for Clinical Trials on Pharmaceutical Products in India and also with the Good Clinical Practice Guidelines issued by the Central Drugs Standard Control Organisation of the Indian Ministry of Health. The study was performed in accordance with the principles stated in the Declaration of Helsinki, and all patients gave written informed consent.

Study design

This 45-days multicenter, open, noncomparative, prospective phase 4 study in an urban primary care setting included consecutive adult outpatients of either sex who were either uncontrolled on CCB monotherapy (≥140/90 mm Hg, or both) or newly diagnosed with grade 2 (SBP 160–179 mm Hg or DBP 100–109 mm Hg) or grade 3 essential hypertension (SBP≥180 or DBP≥100 mm Hg). Patients with a history of hypersensitivity to indapamide or amlodipine, or contraindication to thiazide-like diuretics or CCBs, were excluded from the study. Other exclusion criteria included a recent (within 3 months) history of myocardial infarction or cerebrovascular event; history of heart failure; uncontrolled arrhythmia; uncontrolled diabetes; severe renal dysfunction (estimated glomerular filtration rate [eGFR] <30 mL/min); serious liver disorders; pregnancy; or lactation.

Seven physicians with experience in hypertension management, and adequate clinical and laboratory facilities, recruited hypertensive patients eligible to receive the study medication between April 29 and August 27, 2010, and agreed to implement the study protocol.

Patients previously uncontrolled on CCB monotherapy stopped their previous CCB. All patients received one tablet of single-pill combination indapamide SR/amlodipine 1.5/5 mg in the morning for the next 45 days. Treatment of associated disease was allowed at the discretion of the physician, but concurrent antihypertensive medication was forbidden. Patients were followed up and reassessed after 15, 30, and 45 days, up to the conclusion of the study on October 11, 2010. Laboratory investigations, which included hematology, biochemistry, urinalysis, and electrocardiography, were carried out at the preselection visit and last study visit. At each follow-up visit, BP was measured by mercury sphygmomanometer in the morning, with the patient sitting. The average of 3 readings was recorded. To compare the relative antihypertensive efficacy of indapamide SR/amlodipine in reducing SBP (ΔSBP) versus DBP (ΔDBP), a ΔSBP/ΔDBP ratio from baseline to day 45 was calculated. Patients were also asked open-ended questions about side effects experienced since the previous visit.

The primary outcome was mean BP change from baseline to end. The number of patients achieving BP control (<140/90 mm Hg) was a secondary outcome. Safety and tolerability were also evaluated via reporting of side effects, including pedal edema, and monitoring of laboratory parameters.

Statistical methods

Baseline characteristics are summarized as number of patients and percentage (%) for categorical variables and mean±standard deviation for continuous variables. The analysis was performed on an intention-to-treat basis. The underlying assumption of the statistical analysis was that all variables had a normal probability distribution. Values for baseline BP, end BP, and BP reduction from baseline to days 15, 30, and 45 are presented as means (mm Hg) and corresponding 95% confidence intervals (CI) using a paired t-test. These mean values were used to show the systolic and diastolic BP response to indapamide SR/amlodipine for all hypertensive patients, those previously untreated with grade 2 or grade 3 hypertension, those uncontrolled BP on CCB monotherapy, and diabetes. BP control (<140/90 mm Hg) was summarized as numbers of patients and percentages (%). A paired t-test was used to assess changes in laboratory parameters from baseline to 45 days for significance. Significance was defined as a two-tailed p

value<0.05. Data were analyzed using the statistics program SPSS version 11.

Results

Baseline characteristics are presented in **Table 1**. Mean age of the 196 patients was 52.3 years, just over half (51%) were female, and nearly two-thirds (65%) had grade 2 (n = 115 [59%]) or 3 (n = 12 [6%]) hypertension. Baseline BP in the overall population was 160.2±15.1/97.9±6.8 mm Hg. No patients had severe renal dysfunction (eGFR <30 mL/min). Previously untreated patients constituted over half (n = 108 [55%]) the population, and under half (n = 88 [45%]) were uncontrolled on CCB monotherapy. Thirty-one patients (16%) had diabetes. Over the course of the study, 18 (9%) patients withdrew (lack of efficacy in 1 [<1%], dizziness in 2 [1%], other reasons in 2 [1%], and 13 [7%] lost to follow-up) (**Figure S1**).

Treatment with single-pill combination indapamide SR/ amlodipine reduced overall mean BP by 16.7/10.9 mm Hg after 15 days and by 28.5/15.6 mm Hg at 45 days (**Figure S2 and Table 2**). In patients previously uncontrolled on CCB monotherapy (most commonly amlodipine 5 mg), SBP and DBP fell by 22.0 and 13.1 mm Hg after 45 days. Over the same period, SBP and DBP fell by 33.1 and 18.4 mm Hg in patients with grade 2 hypertension, and by 51.2 and 20.3 mm Hg in patients with grade 3 hypertension. In the overall population, most patients (n = 166 [85%]) achieved BP control (<140/90 mm Hg) after 45 days' treatment (**Figure S3**). By day 45, the percentage of controlled hypertensive patients was 82% (n = 72) in patients previously uncontrolled on CCB monotherapy and 87% (n = 94) in previously untreated patients. In the overall population, ΔSBP/ΔDBP was 1.83 from baseline to day 45. The corresponding ΔSBP/ΔDBP ratios in grade 2 and grade 3 hypertensive patients were 1.80 and 2.52 (**Figure S2**).

Adverse events were reported by 3 (2%) patients. Of these, 2 (1%) experienced dizziness leading to withdrawal, and 1 (<1%) complained of weakness (but completed the study). No other side effects were reported, in particular pedal edema. After 45 days, there were no clinically relevant changes in laboratory parameters versus baseline: plasma fasting glucose, −2.8 mg/dL (p = 0.096); serum sodium, −0.08 mEq/L (p = 0.94); serum potassium, −0.08 mEq/L (p = 0.68); total cholesterol, +1.2 mg/dL (p = 0.58); high-density lipoprotein cholesterol, +0.39 mg/dL (p = 0.74); low-density lipoprotein cholesterol, +1.6 mg/dL (p = 0.43); triglycerides, +7.3 mg/dL (p = 0.03); and no change in serum creatinine (p = 0.89). Most patients (n = 194 [99%]) adhered to treatment.

Table 1. Baseline characteristics of hypertensive patients eligible to receive single-pill combination indapamide SR/ amlodipine 1.5 mg/5 mg.

	N = 196
Demographic characteristics	
Age (years)	52.3±11.4
Sex (female)	99 (51%)
Current smoker	11 (6%)
Body mass index (kg/m^2)	26.1±4.6
Cardiovascular risk	
Systolic blood pressure (mm Hg)	160.2±15.1
Diastolic blood pressure (mm Hg)	97.9±6.8
Coronary artery disease	5 (3%)
TC/HDL ratio	4.2±0.82
Left ventricular hypertrophy	3 (2%)
Diabetes	31 (16%)
Medical history	
Grade 1 hypertension	69 (35%)
Grade 2 hypertension	115 (59%)
Grade 3 hypertension	12 (6%)
Prior antihypertensive treatment	
CCB monotherapy	88 (45%)
Untreated	108 (55%)
Laboratory parameters	
Fasting plasma glucose (mg/dL)	100.8±27.2
Total cholesterol (mg/dL)	180.5±32.0
LDL cholesterol (mg/dL)	105.9±32.3
HDL cholesterol (mg/dL)	43.7±12.1
Triglycerides (mg/dL)	133.3±61.3
Serum sodium (mEq/L)	139.6±10.2
Serum potassium (mEq/L)	4.2±0.5
Serum creatinine (mg/dL)	0.9±0.2
eGFR* (mL/min)	87.8±30.6

Values are means±standard deviation. All other values are numbers and percentages. CCB, calcium channel blocker; eGFR, estimated glomerular filtration rate; HDL, high-density lipoprotein; LDL, low-density lipoprotein; SR, sustained-release; TC, total cholesterol.
*calculated using the 4-variable MDRD formula.
doi:10.1371/journal.pone.0092955.t001

Discussion

Treatment with once-daily indapamide SR/amlodipine 1.5/ 5 mg led to a mean reduction in BP of 28.5/15.6 mm Hg after 45 days and controlled hypertension (BP<140/90 mm Hg) in 85% of the overall population. Response to treatment was similar, regardless of whether patients were previously uncontrolled on CCB monotherapy, untreated, or had a history of diabetes. Treatment was well tolerated, with few patients reporting side effects or discontinuing treatment, and adherence was satisfactory. There were no new cases of pedal edema or hypokalemia. No clinically relevant changes in laboratory parameters were reported.

BP control in our study was initially better in patients uncontrolled on CCB monotherapy than in untreated patients, but the rate of BP control was more rapid in untreated patients so by the end of the study, this situation had reversed. In clinical practice, uncontrolled SBP is largely responsible for the low BP control rate observed [6]. High SBP is more difficult to manage and requires more drug therapy to control than high DBP. The 2013 European guidelines on hypertension management acknowledge the usefulness of both diuretics and CCBs in isolated systolic hypertension by listing them as preferred antihypertensive agents in this condition [13]. Systolic hypertension has also been observed in middle-aged hypertensive patients, in whom it is associated with an increased risk of cardiovascular mortality [22]. Both indapamide SR and amlodipine have been shown to be particularly effective at reducing SBP [17]. The magnitude of the blood pressure reduction seen with indapamide SR/amlodipine in our study was in line with what was expected, considering the two agents separately [17].

Table 2. Baseline and end blood pressure, and blood pressure reduction from baseline in different groups of hypertensive patients.

Blood pressure	SBP (mm Hg)	DBP (mm Hg)
All patients		
Baseline	160.2±15.1	97.9±6.8
End	132.2±9.5	82.4±4.7
Reduction at 15 days	16.7 (14.5 to 18.9)	10.9 (9.7 to 12.1)
Reduction at 30 days	24.3 (22.1 to 26.5)	13.9 (12.7 to 15.1)
Reduction at 45 days	28.5 (26.4 to 30.6)	15.6 (14.5 to 16.7)
Patients previously uncontrolled on CCB monotherapy		
Baseline	153.1±16.2	99.9±6.4
End	130.4±9.5	82.1±5.1
Reduction at 15 days	9.9 (6.6 to 13.2)	7.6 (6.0 to 9.2)
Reduction at 30 days	18.0 (14.9 to 21.1)	10.7 (8.9 to 12.5)
Reduction at 45 days	22.0 (19.3 to 24.7)	13.1 (11.3 to 14.9)
Previously untreated patients		
Baseline	167.3±11.2	101.3±6.0
End	133.6±9.3	82.6±4.4
Reduction at 15 days	22.0 (19.5 to 24.5)	13.5 (12.0 to 15.0)
Reduction at 30 days	29.3 (26.7 to 31.9)	16.4 (14.9 to 17.9)
Reduction at 45 days	33.6 (30.0 to 36.2)	17.6 (16.3 to 18.9)
Patients with grade 2 hypertension		
Baseline	167.6±10.6	100.8±5.0
End	133.2±9.4	82.7±4.1
Reduction at 15 days	20.6 (18.3 to 22.9)	13.6 (12.1 to 15.1)
Reduction at 30 days	29.4 (26.9 to 31.9)	17.0 (15.6 to 18.5)
Reduction at 45 days	33.1 (30.7 to 35.4)	18.4 (17.0 to 19.7)
Patients with grade 3 hypertension		
Baseline	194.3±6.7	102.8±8.7
End	143.2±7.5	81.8±5.7
Reduction at 15 days	34.9 (26.4 to 43.5)	12.2 (6.2 to 18.1)
Reduction at 30 days	47.1 (39.5 to 54.7)	16.3 (11.9 to 20.8)
Reduction at 45 days	51.2 (45.1 to 57.2)	20.3 (13.7 to 26.8)
Diabetic patients		
Baseline	163.5±19.7	96.7±8.7
End	132.1±10.5	82.5±5.3
Reduction at 15 days	11.9 (2.1 to 21.7)	9.8 (6.1 to 13.5)
Reduction at 30 days	24.8 (17.5 to 32.1)	11.0 (7.1 to 14.9)
Reduction at 45 days	31.4 (25.3 to 37.5)	14.2 (10.7 to 17.7)

Values are presented as means and corresponding 95% confidence intervals or standard deviation. CCB, calcium channel blocker; DBP, diastolic blood pressure; SBP, systolic blood pressure.
doi:10.1371/journal.pone.0092955.t002

SBP reduction with indapamide SR/amlodipine compared favorably with that of other antihypertensive single-pill combinations assessed for efficacy and acceptability. Single-pill combinations containing a diuretic and RAAS blocker have ΔSBP/ΔDBP ratios that ranged from 1.34 (−20.3/−15.2 mm Hg with valsartan 160 mg/HCTZ 12.5 mg) to 1.69 (−21.1/−12.5 mm Hg with valsartan 320 mg/HCTZ 25 mg) [23,24], compared with 1.83 (−28.5/−15.6 mm Hg) with indapamide SR 1.5 mg/amlodipine 5 mg. In patients with the severe hypertension (grade 3), the ΔSBP/ΔDBP ratio after 6 weeks with losartan 50 mg/hydrochlorothiazide 12.5 mg was 1.41 (−25.1/−17.8 mm Hg) [23], and 1.37 (−33.2/−24.2 mm Hg) with valsartan 320 mg mg/HCTZ 25 mg [25], compared with 2.52 (−51.2/20.3 mm Hg) with indapamide SR 1.5 mg/amlodipine 5 mg in our study. Comparison with antihypertensive monotherapy showed that BP reduction over 8 to 12 weeks with diuretics was greater than that of other antihypertensive classes: −19.2/−11.1 mm Hg versus −16.4/−11.4 mm Hg with CCBs, −15.6/−10.8 mm Hg with ACE inhibitors, −14.8/−11.4 mm Hg with beta-blockers, −13.5/−11.3 with direct renin inhibitor, and −13.2/−10.3 mm Hg with ARBs [17]. In this meta-analysis, indapamide SR 1.5 mg and amlodipine 5 mg were the best agents in their classes, reducing BP by 22.2/11.7 mm Hg (n = 265) and 19.9/11.5 mm Hg (n = 316), respectively. Although frequently still used in antihypertensive combinations, thiazide diuretics, like HCTZ, are not favored by National Institute of Health and Clinical Excellence hypertension guidelines [26]. These guidelines recommend the agent used in our study, indapamide SR, on the basis of evidence from large outcome trials and indapamide's neutral electrolytic and metabolic effects.

Indapamide SR directly lowers peripheral resistance and has a direct vasorelaxant effect on blood vessels [27,28], which complements the vasodilation produced by amlodipine and enhances overall BP reduction [29,30]. Both drugs control BP over 24 hours [28,31] and have been shown to reduce SBP variability [18]. Diuretic/CCB combinations have also been shown to successfully reduce outcomes in patients with hypertension [14,15,32]. For instance, the incidence of fatal and nonfatal myocardial infarction in VALUE was 19% less with an amlodipine/diuretic regimen than an ARB/diuretic regimen (4.1% vs 4.8%; hazard ratio, 1.19; 95% CI, 1.02 to 1.38; $p = 0.02$) [15]. Our adherence results corroborate previous findings that fixed-dose combination therapy in hypertension is associated with greater adherence to prescribed antihypertensive regimens [33].

The lack of new cases of pedal edema in our study might be explained by indapamide SR [27]. Postcapillary venous relaxation, the result of an indapamide SR-induced decrease in sensitivity of the vasculature to circulating catecholamines, may explain the reduced risk of edema [34]. Furthermore, low-dose CCB, eg, amlodipine 5 mg, has also been shown to be associated with a lower incidence of peripheral edema than high-dose CCB, eg, amlodipine 10 mg [35].

This study had the typical limitations associated with single-arm, open-label studies. The antihypertensive effect of the single-pill combination was not compared with another similar formulation using a randomized protocol. Further results (adjusted for confounding factors) from randomized controlled trials comparing indapamide SR/amlodipine with other treatment options would be useful. Other limitations were a lack of generalizability (due to the observational nature of the study) and possible regression to the mean. We cannot exclude an effect of withdrawals or loss to follow-up on our results (selection bias). Nevertheless, patients received treatment under controlled conditions, and the findings indicate the value of this antihypertensive single-pill combination in clinical practice. Our BP target of <140/90 mm Hg is slightly different from current ESH/ESC guidelines for hypertensive patients with diabetes mellitus, which propose a target of <140/85 mm Hg [13]. However, the optimal BP target for this group of patients is a subject of debate, and our target of <140/90 mm Hg is generally in line with international guidelines [36]. Our definition of severe renal dysfunction does not account for the latest KDIGO (Kidney Disease: Improving Global

Outcomes) guidelines [37]. Long-term benefit was not assessed, but the efficacy and safety of both indapamide SR and amlodipine has been determined in international randomized controlled trials [2,38]. These international trials mitigate the limitation of geographical recruitment in our study, too. Moreover, the study of efficacy at a national level is of interest, as country of birth may influence cardiovascular risk in hypertensive patients [21]. Furthermore, studies of blood pressure variability and pedal edema may help better develop the subject.

Conclusion

In hypertensive patients who required combination treatment—patients previously uncontrolled on CCB monotherapy or untreated with grade 2 or 3 essential hypertension—single-pill combination indapamide SR/amlodipine reduced BP effectively, especially SBP, after 45 days. Indapamide SR/amlodipine was also safe and well tolerated.

Supporting Information

Figure S1 Study flowchart.
(TIFF)

Figure S2 Systolic blood pressure (SBP) and diastolic blood pressure (DBP) response to single-pill indapamide sustained-release/amlodipine in all hypertensive patients (panel A), patients with grade 2 hypertension (panel B), and patients with grade 3 hypertension (panel C) over 45 days. ΔSBP and ΔDBP values are at 45 days.
(TIFF)

Figure S3 Blood pressure control* with single-pill indapamide sustained-release/amlodipine in hypertensive patients previously uncontrolled on CCB monotherapy and previously untreated hypertensive patients. *systolic blood pressure <140 mm Hg, diastolic blood pressure <90 mm Hg, or both. CCB, calcium channel blocker.
(TIFF)

Protocol S1 Trial Protocol.
(PDF)

Checklist S1 CONSORT checklist.
(PDF)

Author Contributions

Conceived and designed the experiments: UJ JH DJN VKG KKT M. Siraj PS M. Safar. Performed the experiments: UJ JH DJN VKG KKT M. Siraj PS. Analyzed the data: UJ JH DJN VKG KKT M. Siraj PS M. Safar. Contributed reagents/materials/analysis tools: UJ JH DJN VKG KKT M. Siraj PS M. Safar. Wrote the paper: UJ JH DJN VKG KKT M. Siraj PS M. Safar.

References

1. Turnbull F (2003) Effects of different blood-pressure-lowering regimens on major cardiovascular events: results of prospectively-designed overviews of randomised trials. Lancet 362: 1527–1535.
2. Beckett NS, Peters R, Fletcher AE, Staessen JA, Liu L, et al. (2008) Treatment of hypertension in Patients 80 years of Age or Older. N Engl J Med 358: 1887–1898.
3. Collins R, Peto R (1994) Antihypertensive drug therapy: effects on stroke and coronary heart disease. In: Swales JD, eds. Textbook of Hypertension. Oxford, UK: Blackwell Scientific Publications; 1156–1164.
4. Mohan V, Deepa M, Farooq S, Datta M, Deepa R (2007) Prevalence, awareness and control of hypertension in Chennai–The Chennai Urban Rural Epidemiology Study (CURES-52). J Assoc Physicians India 55: 326–332.
5. Tocci G, Rosei EA, Ambrosioni E, Borghi C, Ferri C, et al. (2012) Blood pressure control in Italy: analysis of clinical data from 2005–2011 surveys on hypertension. J Hypertens 30: 1065–1074.
6. Williams B, Lindholm LH, Sever P (2008) Systolic pressure is all that matters. Lancet 371: 2219–2221.
7. Corrao G, Nicotra F, Parodi A, Zambon A, Heiman F, et al. (2011) Cardiovascular protection by initial and subsequent combination of antihypertensive drugs in daily life practice. Hypertension 58: 566–572.
8. Egan BM, Bandyopadhyay D, Shaftman SR, Wagner CS, Zhao Y, et al. (2012) Initial monotherapy and combination therapy and hypertension control the first year. Hypertension 59: 1124–1131.
9. Mancia G, De Backer G, Dominiczak A, Cifkova R, Fagard R, et al. (2007) 2007 Guidelines for the management of arterial hypertension: The Task Force for the Management of Arterial Hypertension of the European Society of Hypertension (ESH) and of the European Society of Cardiology (ESC). Eur Heart J 28: 1462–1536.
10. Chobanian AV, Bakris GL, Black HR, Cushman WC, Green LA, et al. (2003) Seventh report of the Joint National Committee on Prevention, Detection, Evaluation, and Treatment of High Blood Pressure. Hypertension 42: 1206–1252.
11. Guide to management of hypertension 2008: assessing and managing raised blood pressure in adults. Updated December 2010. Available: http:/heartfoundation.org.au/SiteCollectionDocument/HypertensionGuidelines2008to2010Update.pdf. Accessed 2-1-22013.
12. Gupta AK, Arshad S, Poulter NR (2010) Compliance, safety, and effectiveness of fixed-dose combinations of antihypertensive agents: a meta-analysis. Hypertension 55: 399–407.
13. Mancia G, Fagard R, Narkiewicz K, Redon J, Zanchetti A, et al. (2013) 2013 ESH/ESC Guidelines for the management of arterial hypertension: The Task Force for the management of arterial hypertension of the European Society of Hypertension (ESH) and of the European Society of Cardiology (ESC). Eur Heart J 34: 2159–2219.
14. Liu L, Zhang Y, Liu G, Li W, Zhang X, et al. (2005) The Felodipine Event Reduction (FEVER) Study: a randomized long-term placebo-controlled trial in Chinese hypertensive patients. J Hypertens 23: 2157–2172.
15. Julius S, Kjeldsen SE, Weber M, Brunner HR, Ekman S, et al. (2004) Outcomes in hypertensive patients at high cardiovascular risk treated with regimens based on valsartan or amlodipine: the VALUE randomised trial. Lancet 363: 2022–2031.
16. Mulatero P, Verhovez A, Morello F, Veglio F (2007) Diagnosis and treatment of low-renin hypertension. Clin Endocrinol (Oxf) 67: 324–334.
17. Baguet JP, Legallicier B, Auquier P, Robitail S (2007) Updated meta-analytical approach to the efficacy of antihypertensive drugs in reducing blood pressure. Clin Drug Invest 27: 735–753.
18. Zhang Y, Agnoletti D, Safar ME, Blacher J (2011) Effect of antihypertensive agents on blood pressure variability: the Natrilix SR versus candesartan and amlodipine in the reduction of systolic blood pressure in hypertensive patients (X-CELLENT) study. Hypertension 58: 155–160.
19. Wang JG, Li Y, Franklin SS, Safar M (2007) Prevention of stroke and myocardial infarction by amlodipine and angiotensin receptor blockers: A quantitative overview. Hypertension 50: 181–188.
20. van Vark LC, Bertrand M, Akkerhuis KM, Brugts JJ, Fox K, et al. (2012) Angiotensin-converting enzyme inhibitors reduce mortality in hypertension : a meta-analysis of randomized clinical trials of renin-angiotensin-aldosterone-system inhibitors involving 158,998 patients. Eur Heart J 33: 2088–2097.
21. Thomas F, Pannier B, Safar ME (2012) Impact of country of birth on arterial function in subjects living in France. J Am Soc Hypertens 6: 405–413.
22. Franklin SS, Jacobs MJ, Wong ND, L'Italien GJ, Lapuerta P (2001) Predominance of isolated systolic hypertension among middle-aged and elderly US hypertensives: analysis based on National Health and Nutrition Examination Survey (NHANES) III. Hypertension 37: 869–874.
23. Salerno CM, Demopoulos L, Mukherjee R, Gradman AH (2004) Combination angiotensin receptor blocker/hydrochlorothiazide as initial therapy in the treatment of patients with severe hypertension. J Clin Hypertens (Greenwich) 6: 614–620.
24. Pool JL, Glazer R, Weinberger M, Alvarado R, Huang J, et al. (2007) Comparison of valsartan/hydrochlorothiazide combination therapy at doses up to 320/25 mg versus monotherapy: a double-blind, placebo-controlled study followed by long-term combination therapy in hypertensive adults. Clin Ther 29: 61–73.
25. Calhoun DA, Glazer RD, Pettyjohn FS, Coenen PD, Zhao Y, et al. (2008) Efficacy and tolerability of combination therapy with valsartan/hydrochlorothiazide in the initial treatment of severe hypertension. Curr Med Res Opin 24: 2303–2311.
26. McManus RJ, Caulfield M, Williams B (2012) NICE hypertension guideline 2011: evidence based evolution. BMJ 344: e181
27. Moore RA, Seki T, Oheim K (1977) Antihypertensive action of indapamide and review of pharmacology and toxicology. Curr Med Res Opin 5: 25–32.
28. Waeber B, Rotaru C, Feihl F (2012) Position of indapamide, a diuretic with vasorelaxant activities, in antihypertensive therapy. Expert Opin Pharmacother 13: 1515–1526.

29. Ferrari R (1997) Major differences among the three classes of calcium antagonists. Eur Heart J 18 Suppl A: A56–A70.
30. Seidel CL, Bohr DF (1971) Calcium and vascular smooth muscle contraction. Circ Res 28: Suppl-95.
31. Mallion JM, Asmar R, Ambrosioni E, Macmahon M, Coupez JM, et al. (1996) [Evaluation of trough:peak ratio of indapamide 1.5 mg sustained-release form assessed by ambulatory blood pressure monitoring]. Arch Mal Coeur Vaiss 89: 27–38.
32. Matsuzaki M, Ogihara T, Umemoto S, Rakugi H, Matsuoka H, et al. (2011) Prevention of cardiovascular events with calcium channel blocker-based combination therapies in patients with hypertension: a randomized controlled trial. J Hypertens 29: 1649–1659.
33. Taddei S (2012) Fixed-dose combination therapy in hypertension: pros. High Blood Press Cardiovasc Prev 19: 55–57.
34. Gustafsson D (1987) Microvascular mechanisms involved in calcium antagonist edema formation. J Cardiovasc Pharmacol 10 Suppl 1: S121–S131.
35. Makani H, Bangalore S, Romero J, Htyte N, Berrios RS, et al. (2011) Peripheral edema associated with calcium channel blockers: incidence and withdrawal rate–a meta-analysis of randomized trials. J Hypertens 29: 1270–1280.
36. Arguedas JA, Leiva V, Wright JM (2013) Blood pressure targets for hypertension in people with diabetes mellitus. Cochrane Database Syst Rev 10: CD008277
37. Wheeler DC, Becker GJ (2013) Summary of KDIGO guideline. What do we really know about management of blood pressure in patients with chronic kidney disease? Kidney Int 83: 377–383.
38. Law MR, Morris JK, Wald NJ (2009) Use of blood pressure lowering drugs in the prevention of cardiovascular disease: meta-analysis of 147 randomised trials in the context of expectations from prospective epidemiological studies. BMJ 338: b1665
